# Autologous Organoid‐T Cell Co‐Culture Platform for Modeling of Immune‐Mediated Drug‐Induced Liver Injury

**DOI:** 10.1002/advs.202508584

**Published:** 2025-09-26

**Authors:** Fadoua El Abdellaoui Soussi, Michael Brusilovsky, Emma Buck, W Clark Bacon, Sina Dadgar, Aaron Fullerton, Victoria Marsh Durban, Riccardo Barrile, Michael A. Helmrath, Takanori Takebe, Adrian Roth, Magdalena Kasendra

**Affiliations:** ^1^ Center for Stem Cell and Organoid Medicine Cincinnati Children's Hospital Medical Center Cincinnati OH 45219 USA; ^2^ Genentech, Inc. 1 DNA Way South San Francisco CA 94080 USA; ^3^ Molecular Devices LLC San Jose CA 95134 USA; ^4^ Department of Biomedical Engineering College of Engineering and Applied Science of the University of Cincinnati Cincinnati OH 45219 USA; ^5^ Division of Gastroenterology Hepatology and Nutrition & Division of Developmental Biology Cincinnati Children's Hospital Medical Center Cincinnati OH 45229 USA; ^6^ Premium Research Institute for Human Metaverse Medicine (WPI‐PRIMe), and Division of Stem Cell and Organoid Medicine The University of Osaka Suita Osaka 565–0871 Japan; ^7^ F. Hoffmann‐La Roche Ltd, Product Development, Precision Safety Roche Innovation Centre Grenzacherstrasse 124 Basel 4070 Switzerland

**Keywords:** CD8⁺ T cells, HLA‐B*57:01, immune‐mediated toxicity, liver organoids, organoid‐immune co‐culture platforms

## Abstract

Modeling adaptive immune responses in induced pluripotent stem cell (iPSC)‐derived liver systems remains a critical barrier for studying immune‐mediated hepatic diseases, including idiosyncratic drug‐induced liver injury (iDILI). Conventional hepatotoxicity models lack the components required to capture patient‐specific, T cell‐mediated injury. Here, a scalable and matrix‐free human liver organoid (HLO) microarray platform is presented that enables controlled co‐culture of Human Leukocyte Antigen (HLA)‐genotyped, iPSC‐derived HLOs with autologous CD8⁺ T cells. This immune‐competent system supports antigen‐specific T cell activation and reproduces cytotoxic effector responses in a genetically defined context. As a proof‐of‐concept, the platform models clinically relevant iDILI caused by flucloxacillin in HLA‐B*57:01 carriers, recapitulating CD8⁺ T cell proliferation, hepatocyte apoptosis, and variability in immune responses across donors. The system captures hallmark features of adaptive immune‐mediated hepatotoxicity, including secretion of tumor necrosis factor‐alpha and Granzyme B, and cytokeratin‐18 release from injured hepatocytes. By linking genetic susceptibility with functional immune outcomes, this platform provides a modular and scalable approach for evaluating immune‐mediated toxicities. The method offers broad utility for mechanistic studies of drug hypersensitivity, immune‐related adverse events, and preclinical safety assessment in support of precision medicine.

## Graphical abstract

A matrix‐free human liver organoid–T cell co‐culture platform enables modeling of immune‐mediated drug‐induced liver injury (iDILI). Using flucloxacillin and patient‐matched cells, this system recapitulates HLA‐B*57:01–restricted CD8⁺ T cell activation and hepatocyte damage. By linking genetic risk to immune function, this approach enables mechanistic dissection and functional prediction of idiosyncratic hepatotoxicity in vitro.

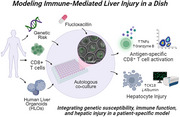

## 1 Introduction

Modeling adaptive immune responses in vitro remains a critical unmet need in liver research and drug development. Immune‐mediated liver injuries – including autoimmune hepatitis, drug hypersensitivity reactions, and immune‐related adverse events from cancer immunotherapies – are driven by complex, patient‐specific mechanisms that current in vitro systems fail to recapitulate. Among these, idiosyncratic drug‐induced liver injury (iDILI) presents a particularly intractable challenge: it is a leading cause of drug withdrawals and regulatory warnings, and accounts for a significant portion of acute liver failure cases unrelated to overdose. These outcomes often occur despite favorable safety profiles in standard preclinical models, highlighting a persistent translational gap between existing in vitro platforms, animal studies, and the human immune responses that ultimately drive patient outcomes.^[^ ^1^, ^2^ ^]^

This gap arises in part from the limitations of conventional hepatotoxicity assays, which rely on direct measures of cytotoxicity and lack the antigen presentation machinery and T cell compartments required to model Human Leukocyte Antigen (HLA)‐restricted, cytotoxic CD8⁺ T cell responses – mechanisms known to be underlie iDILI in clinical settings.^[^ ^3^, ^4^ ^]^ While recent advances in iPSC‐derived hepatocyte systems and liver‐on‐chip technologies have improved physiological fidelity for hepatic metabolism and innate immunity,^[^ ^5^, ^6^ ^]^ they still fail to recapitulate the antigen specificity, T cell priming, and adaptive effector functions essential for modeling immune‐mediated hepatotoxicity. Notably, hepatocyte death in iDILI is not triggered directly by the drug, but by CD8⁺ T cells activated through HLA‐restricted pathways – a mechanism that current models fail to represent.^[^ ^7^, ^8^ ^]^ This mirrors the immune‐mediated injury seen in viral hepatitis, where adaptive immune responses, not the pathogen itself, drive tissue damage, further underscoring the need for models that replicate these dynamics.^[^ ^9^ ^]^ In addition, reliance on primary cells or Matrigel scaffolds hampers scalability, standardization, and the incorporation of genetic diversity—factors critical for investigating patient‐specific risk. To overcome these barriers, we developed a fully human, matrix‐free liver organoid (HLO) microarray platform that integrates iPSC‐derived hepatocytes with autologous CD8⁺ T cells in a genetically defined context. This scalable co‐culture system enables controlled, reproducible assessment of antigen‐specific immune responses and immune‐mediated hepatotoxicity.

As a proof‐of‐concept, we interrogated the platform with flucloxacillin, a clinically relevant and mechanistically well‐characterized example of HLA‐B*57:01‐linked, T cell‐mediated hepatotoxicity.^[^ ^10^, ^11^, ^12^, ^13^, ^14^ ^]^ Despite the strong genetic association, most carriers of the risk allele do not develop liver injury, highligting the limitations of genetic screening alone and the need for functional assays that capture donor‐specific immune activation. Our platform faithfully recapitulates key features of the immune response, including CD8⁺ T cell activation, cytokine secretion, and hepatocyte apoptosis, without relying on supra‐physiological stimuli or surrogate animal models.^[^ ^3^, ^4^, ^7^, ^12^, ^15^, ^16^ ^]^ By bridging the gap between genetic susceptibility and functional immune outcomes, this system provides a broadly applicable tool for studying immune‐mediated toxicities, validating HLA‐associated risks, and enabling precision safety assessment in drug development.

## 2 Results

### 2.1 Matrix‐Free HLO Microarrays Enable Uniform and Scalable Culture Conditions

**Figure 1:**
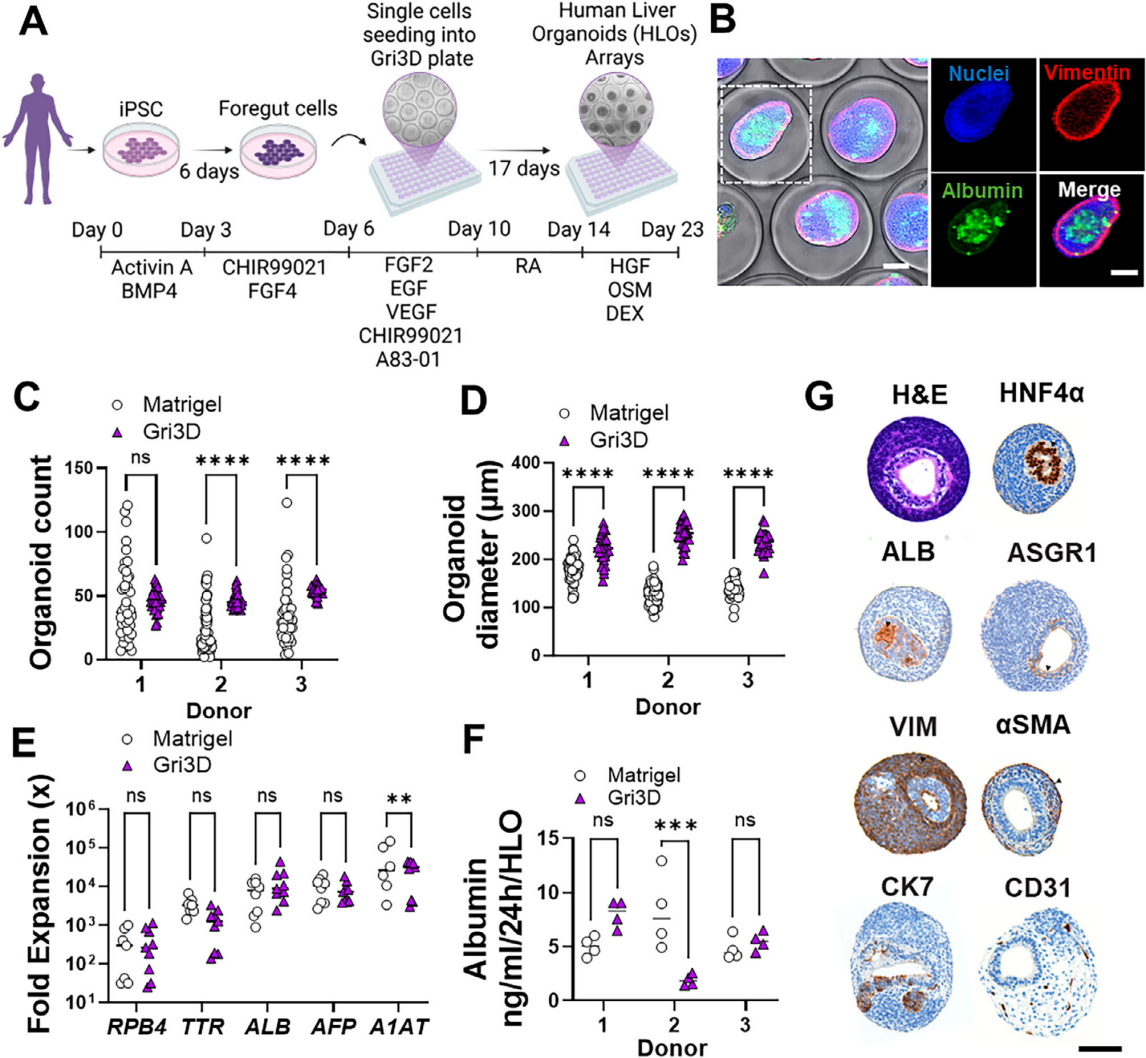
Scalable and Matrix‐Free HLO Microarrays Enable Uniform and Reproducible Culture. A) Schematic overview of the generation of HLO microarrayed cultures from iPSCs. B) Representative image of Day 23 HLOs in a microarray format, showing albumin‐positive hepatocytes surrounded by vimentin‐positive mesenchymal cells. Scale bar, 100 µm. C,D) Quantification of the organoid count (C) and organoid diameter (D) on day 23 in HLO microarrays (Gri3D) versus Matrigel‐grown HLOs across three donors. Each data point represents an individual organoid; n  =  32‐36, ****P < 0.0001; ANOVA, Sidak's post hoc test. E) Expression of early and late (developmentally) hepatocyte markers at day 23 relative to D6. (mean ± SEM, n = 3 biological replicates) ns = non‐significant, *P* > 0.05; (two‐way ANOVA). F) Albumin secretion levels in HLOs grown on Gri3D or embedded in Matrigel from three donors, each data point represents one technical replicate (n = 4); Two‐way ANOVA ***, P ≤ 0.0001 G) Immunohistochemistry characterization of HLO microarrays, showing expression of hepatic (HNF4α, ALB, ASGR1), mesenchymal (VIM, αSMA), endothelial (CD31), and cholangiocyte (CK7) markers. See also Figure S1 (Supporting Information).

We applied an innovative bioengineering approach to generate highly uniform and reproducible HLOs using forced aggregation of iPSC‐derived posterior foregut cells within micropatterned hydrogels (the Gri3D system). These hydrogels were fabricated with 500‐µm microcavities positioned at the bottom of 96‐well plate wells (**Figure** **1**A). Enzymatically dissociated single cells obtained from a foregut monolayer were seeded onto the microcavities to achieve aggregates of ≈250 cells per microcavity. Stepwise liver organoid differentiation was driven by a predefined growth factor regimen incorporated into the culture media according to previously established protocols.^[^ ^6^ ^]^ Organoid formation and maturation were driven by a combination of small molecules and growth factors, yielding three‐dimensional (3D) organoids with functional hepatocytes surrounded by mesenchymal cells, as confirmed by albumin and vimentin staining (Figure 1B).

Comparative analysis with standard Matrigel dome cultures across three iPSC lines demonstrated that the Gri3D system significantly improved homogeneity of the cell culture. HLOs formed at predefined positions with high reproducibility across donors, exhibiting consistent organoid formation efficiency and uniform diameters (Figure 1C,D; Figure S1A, Supporting Information). The coefficient of variation for intra‐ and inter‐donor organoid numbers and diameters was significantly lower than in Matrigel‐based cultures (Figure S1B,C, Supporting Information).

Functional and structural analyses confirmed successful maturation of HLOs within the microarrays. RT‐qPCR‐based gene expression profiling revealed strong expression of key liver maturation markers (*RBP4, AFP, TTR*, *A1AT* and *ALB*) at levels comparable to Matrigel‐based organoids (Figure 1E). Histological analysis validated the presence of key hepatic cell types, including hepatocytes (HNF4α, ALB, ASGR1), hepatic stellate cells (αSMA), cholangiocytes (CK7), and vascular cells (CD31) (Figure 1G). ELISA confirmed comparable albumin secretion to conventional methods (Figure 1F).

In summary, we developed a scalable, matrix‐free, and modular HLO microarray system using Gri3D platform. This approach enhances organoid formation homogeneity while maintaining cellular maturation, structural integrity, and functional characteristics comparable to standard Matrigel‐based cultures.

### 2.2 HLO Microarrays Accurately Capture Intrinsic but Not Immune‐Mediated Hepatotoxicity

To assess the ability of the HLO microarray platform to model both intrinsic and idiosyncratic DILI, we tested its response to two well‐characterized compounds: Chlorpromazine, a drug known to cause dose‐dependent intrinsic hepatotoxicity,^[^ ^17^ ^]^ and Flucloxacillin, which induces iDILI through immune‐mediated mechanisms that require adaptive immune activation.^[^ ^11^, ^14^ ^]^

**Figure 2:**
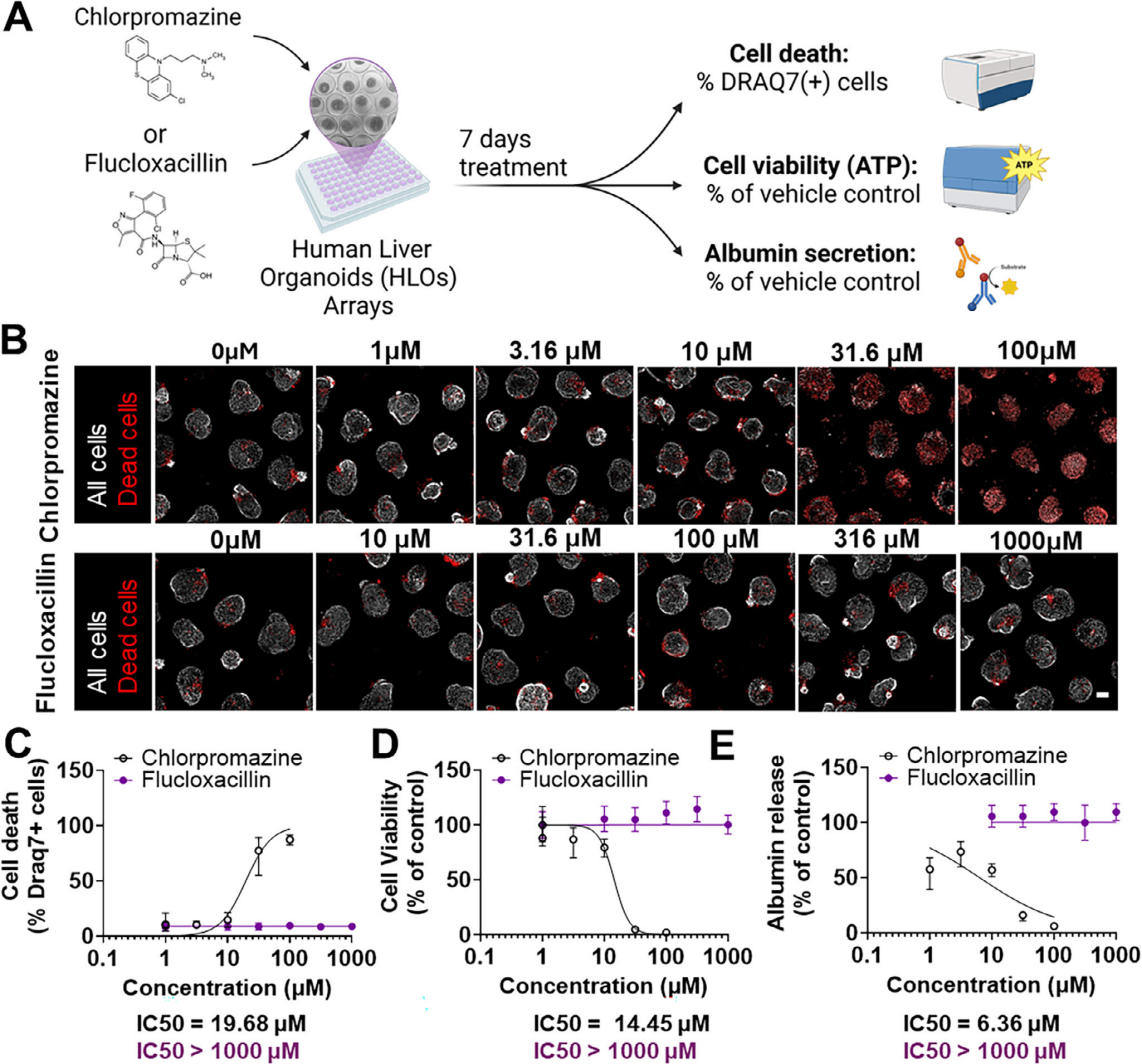
Flucloxacillin Does Not Induce Direct Hepatotoxicity in HLO Microarrays. A) Experimental workflow schematic. B) Representative images of HLO microarrays treated for seven days with Chlorpromazine or Flucloxacillin at different concentrations. Scale bar, 100 µm. C–E) Quantification of cell death (% DRAQ7‐positive cells) (C), cell viability (% relative to the viability of vehicle‐treated controls) (D), and albumin secretion (% relative to the release from vehicle‐treated controls) (E) following treatment with Chlorpromazine (open black circles) or Flucloxacillin (closed purple circles) across five concentrations (n = 8 technical replicates). Data represent mean ± SEM. Representative of three biologically independent experiments is shown, each using HLOs derived from independent differentiations of the same iPSC donor line.

HLO microarrays were exposed to seven‐day repeated‐dose treatment of Chlorpromazine and Flucloxacillin across five half‐log‐spaced concentrations, with vehicle‐treated wells serving as controls. Following treatment, cell viability was assessed by quantifying total cellular ATP content using the CellTiter‐Glo assay, while cytotoxicity was determined by counting DRAQ7‐positive nuclei (dead cells) in organoids. Hepatic functionality was further evaluated by measuring albumin secretion in the culture supernatant via ELISA (**Figure** **2**A).

As expected, Chlorpromazine induced significant dose‐dependent hepatotoxicity, evidenced by marked increase in dead cell counts (DRAQ7), reduced ATP levels, and decreased albumin secretion – collectively indicating loss of viability and impaired liver function (Figure 2B,C). The IC_50_ for ATP‐based viability was 14.4 µM, consistent with prior 3D liver models.^[^ ^18^, ^19^, ^20^ ^]^ Relative to its reported clinical Cmax of ≈0.94 µM,^[^ ^18^ ^]^ this corresponds to a margin of safety (MOS) of ≈15.3, indicating toxicity occurs at supratherapeutic but pharmacologically relevant concentrations commonly used in preclinical safety evaluation.

In contrast, Flucloxacillin showed no evidence of cytotoxicity or functional impairment across all tested concentrations up 1 mM – more than 30‐fold higher than its clinical Cmax (≈31 µM).^[^ ^21^ ^]^ Both viability and albumin secretion remained comparable to vehicle controls throughout (Figure 2B,C). These results align with Flucloxacillin's known immune‐mediated hepatotoxicity in vivo and confirm that the HLO microarray platform does not generate false‐positive signals in the absence of adaptive immune components.

To further assess the robustness and donor‐specific responses, Chlorpromazine was tested across three independent iPSC‐derived HLO lines. It consistently induced dose‐dependent toxicity in all donors (average IC_50_  =  11.12 µM), though the degree of susceptibility varied (CV  =  40.3%), reflecting inter‐individual differences in metabolism and drug response (Figure S2A, Supporting Information). In contrast, Flucloxacillin and Streptomycin – a clinically non‐hepatotoxic antibiotic – elicited no cytotoxicity at clinically relevant exposures (Cmax ≈31 and ≈72.2 µM,^[^ ^22^ ^]^ respectively) (Figures S2B,C, Supporting Information). Streptomycin‐induced reductions were only observed at supra‐therapeutic concentrations (average IC_50_  =  341 µM; CV  =  15.7%), consistent with findings from other 3D liver models that similarly report no hepatotoxicity at clinically relevant doses^[^ ^18^ ^]^ (Figure S2C, Supporting Information).

Collectively, these findings demonstrate that the HLO microarray platform reliably captures intrinsic hepatotoxicity, resolves inter‐donor variability, and exhibits high specificity by showing minimal response to non‐toxic compounds – consistent with prior results using Matrigel‐based HLOs.^[^ ^6^ ^]^ The absence of Flucloxacillin‐induced toxicity, despite its known immune‐mediated effects in vivo, underscores the importance of incorporating immune‐competent co‐culture systems to model adaptive immune mechanisms and fully recapitulate the spectrum of DILI phenotypes

### 2.3 Integration of Autologous CD8^+^ T Cells Establishes Immune Competence in HLO Co‐Cultures

**Figure 3:**
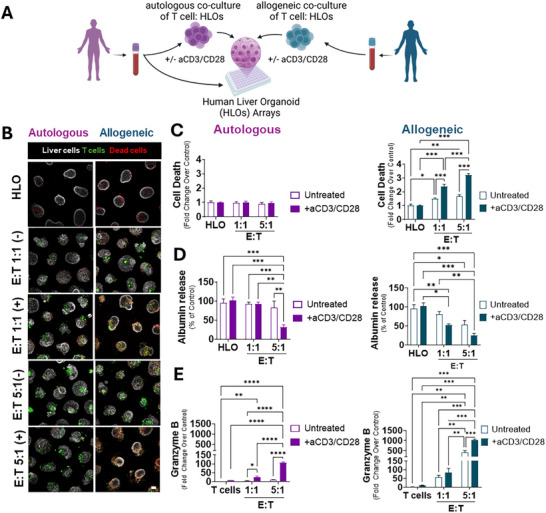
Integration of Autologous CD8^+^ T Cells Establishes Immune Competence in HLO Co‐Cultures. A) Schematic overview of the experimental workflow. B) Representative images of microarrayed HLO and their co‐cultures with autologous or allogeneic CD8^+^ T cells at different effector‐to‐target (E:T) ratios, with (+) or without (‐) anti‐CD3/CD28 stimulation. Scale bar, 100 µm. C–E) Quantification of cell death (C), albumin release (D), and Granzyme B secretion (E) across different mono and co‐culture conditions. *P ≤ 0.05, **P ≤ 0.01, ***P ≤ 0.001, ANOVA and Tukey's tests. Only statistically significant (*P* ≤ 0.05) comparisons are shown. Data represent mean ± SEM, n = 3.

To evaluate whether patient‐matched T cells could enhance immune competence in HLO microarrays and more accurately model iDILI mechanisms, we developed an autologous co‐culture system using naïve CD8^+^ T cells derived from the same donor as the HLOs. This model was compared to an allogeneic co‐culture system, where HLOs were paired with CD8^+^ T cells from a genetically distinct donor, introducing an HLA mismatch (**Figure** **3**A).

Pilot studies were conducted to optimize assay conditions, including media composition to preserve hepatic functions (e.g., albumin secretion) while supporting immune cell activity (e.g., IFN‐γ and Granzyme B release upon stimulation). Additionally, the optimal co‐culture duration was determined (data not shown).

On day 23 of organoid growth, CFSE‐labeled autologous or allogeneic CD8^+^ T cells were added at varying effector‐to‐target (E:T) ratios. Co‐cultures were established with or without T cell receptor (TCR) co‐stimulation using anti‐CD3 and anti‐CD28 antibodies. Organoid viability was assessed via DRAQ7 staining, and immune‐mediated cytotoxicity was quantified by counting CFSE‐negative (HLO) DRAQ7‐positive (dead) cells.

In allogeneic co‐cultures, a significant level of cell death was observed at both 1:1 and 5:1 E:T ratios, particularly under TCR co‐stimulation (Figure 3B,C). Hepatocyte function, assessed by albumin secretion, was significantly impaired in allogeneic co‐cultures upon TCR co‐stimulation at both E:T ratios (Figure 3D). Under unstimulated conditions, albumin secretion was only reduced at the higher E:T ratio (5:1), suggesting that a larger T‐cell population is required to elicit cytotoxic responses driven solely by HLA mismatch. Additionally, Granzyme B release was significantly elevated at 5:1 E:T ratios in both unstimulated and anti‐CD3/CD28‐treated allogeneic co‐cultures but remained undetectable at 1:1, indicating that T‐cell activation and cytotoxicity in allogeneic settings are dose‐dependent and enhanced by TCR co‐stimulation.

Conversely, autologous co‐cultures exhibited no significant cell death, regardless of the E:T ratio or TCR co‐stimulation (Figure 3B,C). However, a decrease in albumin secretion was observed at the 5:1 E:T ratio upon TCR co‐stimulation with anti‐CD3/CD28, likely due to expected non‐specific T cell activation and subsequent Granzyme B release, leading to hepatocyte dysfunction. Notably, albumin levels remained unchanged in unstimulated autologous conditions (Figure 3D,E).

These findings demonstrated the potential of the HLO‐CD8+ T cell co‐culture system for modeling immune‐mediated liver injury while capturing patient‐specific immune responses. Additionally, the autologous nature of the platform minimizes non‐specific T cell activation, reducing the risk of unintended cytotoxic effects, thereby providing a physiologically relevant model for studying iDILI.

### 2.4 HLA‐B*57:01‐Restricted CD8⁺ T Cell Activation in Response to Flucloxacillin

To evaluate the clinical relevance of our platform, we modeled Flucloxacillin‐induced drug‐induced liver injury (DILI) using patient‐derived cells from HLA‐B*57:01 carriers and non‐carriers. Flucloxacillin is a β‐lactam antibiotic, and a well‐established cause of CD8^+^ T cell‐driven immune‐mediated liver injury, predominantly affecting HLA‐B*57:01‐positive individuals.^[^ ^10^, ^11^, ^12^, ^13^ ^]^ However, the precise mechanisms underlying this HLA‐restricted toxicity remain poorly understood.

**Figure 4:**
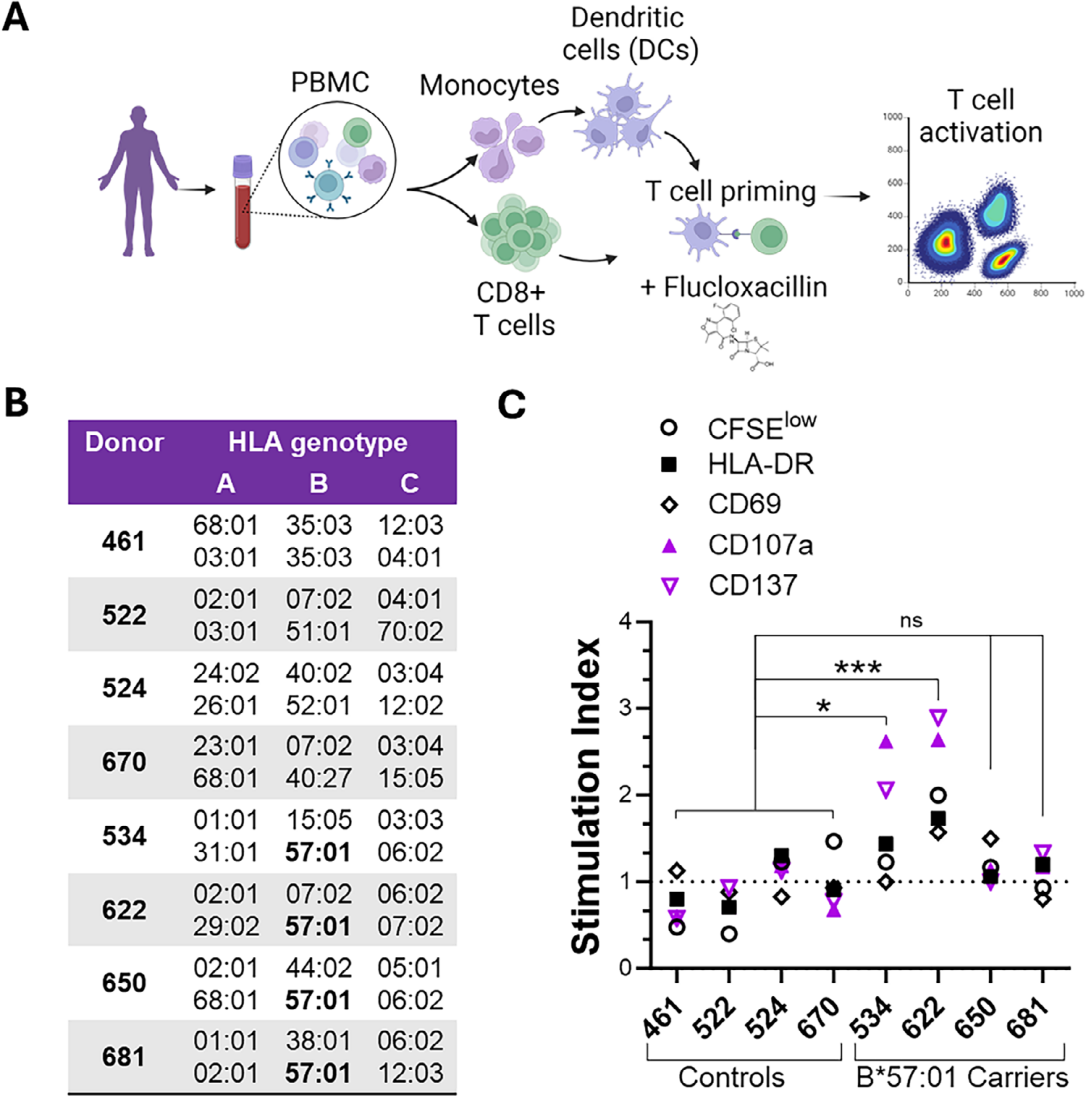
HLA‐B*57:01–Restricted CD8⁺ T Cell Activation by Flucloxacillin Validated Using Dendritic Cell Priming. A) Schematics of the experimental workflow followed in this study. B) HLA class I genotypes of healthy donors, classified as HLA‐B*57:01 carriers and non‐carriers (controls). C) Stimulation index of CD8⁺ T cell activation markers following Flucloxacillin priming in control and HLA‐B*57:01 carrier groups. *P ≤ 0.05, ***P ≤ 0.001, ANOVA and Tukey's tests versus average marker expression in the non‐carrier control group. Representative of three independent assay repeats. See also Figure S3 (Supporting Information).

Peripheral blood samples were collected from four HLA‐B*57:01 carriers and four HLA‐B*57:01 non‐carrier donors (**Figure** **4**B). To generate antigen‐specific CD8⁺ T cells, we employed a monocyte‐derived dendritic cell (mDC) priming assay (Figure 4A). Naïve CD8⁺ T cells and monocytes were isolated from peripheral blood mononuclear cells (PBMCs) of each donor. mDCs were differentiated using IL‐4 and GM‐CSF, followed by maturation and activation with LPS and IFN‐γ. Mature mDCs were then loaded with Flucloxacillin or media alone and used to prime naïve CD8⁺ T cells. After priming, CD8⁺ T cells (i.e., Flucloxacillin‐primed CD8^+^ T cells) were expanded in the presence of a low dose of IL‐15 for 10 days, followed by CFSE labeling and re‐stimulation with freshly loaded mDCs under the same conditions. Flow cytometry was used to assess CD8⁺ T cell maturation, proliferation, and post‐priming activation.

All four HLA‐B57:01‐positive donors exhibited some degree of response to Flucloxacillin, however, only two donors demonstrated a robust over 2‐fold increase in T cell proliferation (CFSE^low^ cells) and activation marker expression relative to media‐alone priming. Specifically, Flucloxacillin priming led to an increase in CD45RO^+^ memory CD8^+^ T cell population, expressing HLA‐DR (activation), CD69 (early activation), and CD137 (antigen‐driven activation). Additionally, increased surface expression of CD107a, a marker of cytotoxic degranulation, indicated specific functional activation of CD8⁺ T cells in response to Flucloxacillin exposure (Figure 4C; Figure S3, Supporting Information).

In contrast, HLA‐B*57:01 non‐carrier donors did not exhibit a comparable response, reinforcing the HLA‐restricted nature of Flucloxacillin‐induced immune activation. These findings highlight the utility of our platform in recapitulating patient‐specific drug‐induced immune responses.

### 2.5 Organoid–T Cell Co‐Culture System Recapitulates HLA‐B*57:01–Restricted Liver Injury Induced by Flucloxacillin

**Figure 5:**
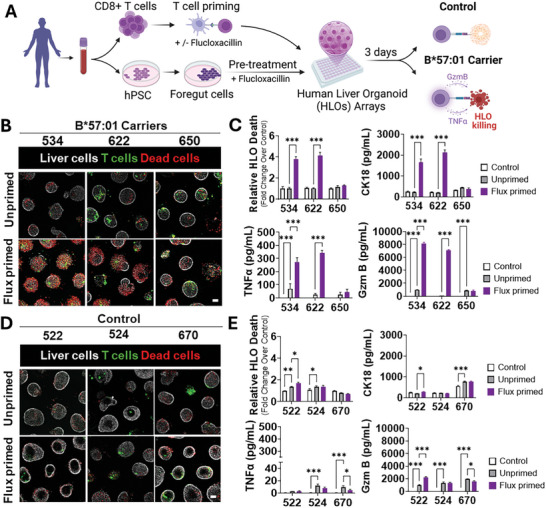
Organoid–T Cell System Recapitulates HLA‐B*57:01–Linked Hepatotoxicity Driven by Flucloxacillin‐Primed CD8⁺ T Cells A) Schematic overview of the experimental workflow. B and D) Representative images of HLO‐CD8^+^ T cell co‐cultures from HLA‐B*57:01 non‐carriers (B) and HLA‐B*57:01 carriers (D) after three days of co‐culture at a 5:1 effector‐to‐target ratio. Culture conditions: unprimed (HLO + unprimed CD8^+^ T cells), flux‐primed (HLO + Flucloxacillin‐primed CD8^+^ T cells). Scale bar, 100 µm. C and E) Quantification of HLO death, cytokeratin‐18 (CK18) release, TNFα, and Granzyme B (GzmB) secretion across experimental conditions.: *P ≤ 0.05, **P ≤ 0.01, ***P ≤ 0.001, ANOVA and Dunnett's tests. Only statistically significant (*P* ≤ 0.05) comparisons are shown.  Data represent mean ± SEM, n = 5.

To model immune‐mediated liver injury, Flucloxacillin‐primed CD8⁺ T cells from three HLA‐B*57:01 carrier and three HLA‐B*57:01 non‐carrier donors were co‐cultured with autologous HLOs pre‐treated with Flucloxacillin (**Figure** **5**A). Co‐cultures with unprimed CD8⁺ T cells served as controls to exclude non‐specific T cell reactivity. HLOs were generated from iPSC lines derived from donor PBMCs and exposed to 100 µM Flucloxacillin for 72 h before initiating co‐culture. On day 23 of maturation, HLOs were co‐cultured with unprimed or Flux‐primed CD8⁺ T cells to assess immune‐mediated hepatocyte injury. Liver damage was quantified using immunofluorescence‐based detection of DRAQ7⁺ dead cells and measurement of cytokeratin‐18 (CK‐18) release, a highly specific biomarker of early‐stage DILI. CK‐18, an intermediate filament protein expressed in hepatocytes and cholangiocytes but absent in immune cells, provided greater specificity for hepatocyte death than traditional markers such as LDH or ATP.

Flucloxacillin‐primed CD8⁺ T cells from two HLA‐B*57:01 carrier donors (534 and 622; robust responders – see Figure 4C), induced significant HLO injury, as evidenced by a 4‐fold increase in DRAQ7⁺ cell death (compared to T cell‐free controls; Figure 5B,C) and significantly elevated CK‐18 release. This cytotoxicity signal correlated with increased secretion of TNF‐α and Granzyme B, known key mediators of iDILI pathogenesis. In contrast, Flucloxacillin‐primed T cells from the other two HLA‐B*57:01 carrier donors (weak responders) and all HLA‐B*57:01 non‐carrier donors (see Figure 4C) failed to induce hepatocyte death or CK‐18 release (Figure 5D,E).

## 3 Conclusion 

The development of in vitro platforms that recapitulate key components of adaptive immune responses – particularly antigen‐specific T cell activation and effector function – in a genetically defined, human‐relevant context remains a longstanding challenge in toxicology and regenerative medicine. Our immune‐competent, matrix‐free liver organoid–T cell co‐culture platform addresses this need by enabling systematic evaluation of HLA‐restricted, CD8⁺ T cell–mediated hepatotoxicity in a fully autologous and scalable system. Unlike previous models that rely on indirect cytokine stimulation or conditioned media transfer, our approach supports direct, antigen‐specific interactions between primed T cells and hepatocytes under physiologically relevant, defined, and reproducible conditions.^[^ ^8^, ^11^, ^23^ ^]^

This methodological advancement confers several key advantages. First, it enables functional modeling of genetic risk alleles – such as HLA‐B*57:01 – within a clinically relevant and heterogeneous context. Second, the use of micropatterned hydrogel arrays and matrix‐free differentiation supports scalable, uniform organoid formation compatible with high‐content imaging and functional analysis.^[^ ^24^ ^]^ In contrast to conventional organoid models that rely on Matrigel – a murine‐derived basement membrane matrix known to hinder immune cell function^[^ ^25^ ^]^ – our matrix‐free design enables unrestricted T cell–hepatocyte interactions in a physiologically relevant environment. This improves immune compatibility and renders the platform particularly well‐suited for modeling antigen‐specific responses in the context of immunotoxicity. Third, the incorporation of clinically relevant readouts – including cytokeratin‐18 release, TNF‐α and Granzyme B secretion, and DRAQ7⁺ hepatocyte death – captures key mechanisms of CD8⁺ T cell‐mediated liver injury.^[^ ^26^ ^]^

Our matrix‐free HLO microarray platform demonstrated high specificity for intrinsic hepatotoxicants, such chlorpromazine, while remaining unresponsive – at clinically relevant concentrations – to compounds like streptomycin and flucloxacillin, which lack direct cytotoxicity. These results were consistent across genetically distinct iPSC donors, confirming reproducibility and the ability to resolve inter‐individual variation in DILI susceptibility. This highlights the system's robustness in modeling intrinsic DILI, as previously shown using Matrigel‐based HLOs in a high‐throughput screen of 238 marketed drugs.^[^ ^6^ ^]^ Importantly, the matrix‐free configuration enables immune modelling via the controlled addition of adaptive immune components.

By incorporating autologous CD8⁺ T cells, the platform gains immune competence and becomes capable of modeling idiosyncratic, HLA‐restricted liver injury. In this proof‐of‐concept study, we have focused on flucloxacillin‐induced hepatotoxicity observed clinically in HLA‐B*57:01 carriers. Our data showed that CD8⁺ T cells from HLA‐B*57:01‐positive – but not ‐negative – donors could be primed by flucloxacillin to acquire an effector phenotype, evidenced by a twofold increase in HLA‐DR, CD137, CD69, and CD107a expression. Upon co‐culture with autologous liver organoids, primed T cells from strong responders triggered significant hepatocyte injury, marked by a fourfold increase in DRAQ7⁺ cell death, elevated CK‐18 release, and enhanced secretion of Granzyme B and TNF‐α. In contrast, CD8⁺ T cells from weak responders and all non‐carriers failed to elicit cytotoxic responses in co‐culture. These findings mirror clinical variability in flucloxacillin‐induced DILI and reinforce the value of patient‐specific, functional in vitro models.^[^ ^10^, ^12^ ^]^

This dual capability – modeling both antigen‐specific CD8⁺ T cell activation and subsequent hepatocyte injury in a controlled, autologous in vitro setting – positions our system as a powerful tool for linking immune activation to hepatotoxic outcomes. The observed inter‐individual variability also highlights that genetic predisposition alone is insufficient to predict adverse outcomes, emphasizing the need for functional immunotoxicology platforms that support patient‐specific risk assessment.^[^ ^11^, ^23^ ^]^

Beyond flucloxacillin, the modular design of this platform enables broad application to other forms of immune liver injury – including hepatitis triggered by checkpoint inhibitors, autoimmune liver disease, or biologic drug immunogenicity. Its autologous configuration further supports the dissection of inter‐individual variability in T cell priming, effector function, and hepatocyte susceptibility – critical contributors to the pathogenesis of idiosyncratic drug toxicity.

Importantly, this study provides foundational evidence that immune‐mediated DILI phenotypes can be recapitulated in vitro using genetically defined liver organoids and autologous T cells. We acknowledge the limitation of using only flucloxacillin but emphasize that this choice was deliberate to establish feasiblity with a clinically validated test case and define key principles for future expansion.

This platform offers a much‐needed infrastructure for immunotoxicology, combining physiological relevance, genetic specificity, and functional resolution. As the field advances toward mechanism‐based, patient‐relevant safety models, such systems will be essential for early immunogenicity risk assessment and mechanistic discovery.

Future work will focus on scaling the platform through automation, expanding donor diversity to support population‐level analyses, and incorporating single‐cell profiling to reveal transcriptional signatures associated with immune response heterogeneity. Additionally, the integration of iPSC‐derived T cells may further extend the platform's utility by offering a renewable, standardized immune component suitable for higher‐throughput applications.

In summary, we present a scalable, modular, and autologous liver organoid – T cell co‐culture platform for modeling immune‐mediated hepatotoxicity. By integrating genetic risk with functional immune assessment, this system addresses a critical gap in preclinical drug safety and offers a translationally relevant framework for mechanistic discovery and patient‐specific toxicity profiling.

## 4 Experimental Section

### iPSC Cell Lines and Cell Culture

The human iPSC lines used in this study are summarized in Table S1 (Supporting Information). Peripheral blood mononuclear cells (PBMCs) were collected from healthy donors with informed written consent in compliance with institutional ethics guidelines and according to protocols approved by the Institutional Review Board, Cincinnati Children's Hospital Medical Center (IRB approval #2017‐2011). These cells were reprogrammed into iPSCs by the CCHMC Pluripotent Stem Cell Facility using the CytoTune‐iPS 2.0 Sendai Reprogramming Kit (Thermo Fisher Scientific). Upon establishment, all human iPSC lines underwent comprehensive quality control following ISCCR and were routinely authenticated every 10 passages.^[^ ^27^ ^]^ Routine quality control assessments included karyotype analysis, short tandem repeat (STR) profiling, pluripotency marker expression, and mycoplasma contamination testing. iPSC expansion and maintenance were performed as previously described.^[^ ^28^ ^]^ Briefly, undifferentiated iPSCs were cultured on Laminin‐iMatrix‐511 silk‐coated dishes (Nacalai) in mTSER complete medium (STEMCELL Technologies) supplemented with 10 uM Rock inhibitor Y27632 (Tocris). Cells were maintained at 37 °C in 5% CO_2_ and 95% air.

### HLA Typing

Genomic DNA was isolated from the isolated PBMCs using the Qiagen EZ2 Connect instrument with Qiagen blood cartridges. DNA concentration and purity were assessed using a spectrophotometer. High‐resolution HLA typing was performed exclusively by Next Generation Sequencing (NGS) using the One Lambda AllType NGS 11‐Loci kit (Thermo Fisher Scientific) according to the manufacturer's protocol. This kit targets amplification of 11 HLA loci (HLA‐A, ‐B, ‐C, ‐DRB1, ‐DRB3/4/5, ‐DQB1, ‐DPA1, ‐DPB1, and ‐DQA1). Following amplification and library preparation, libraries were sequenced on an Illumina MiSeq instrument. Raw sequencing reads were analyzed using TypeStream Visual NGS Analysis Software (One Lambda, Thermo Fisher Scientific) to align reads to the IMGT/HLA database and assign high‐resolution HLA allele types. All reported high‐resolution HLA types adhere to the Common and Well‐Documented (CIWD) allele definitions version 3.0.0. As parents' samples were not available, haplotypes were not obtained by segregation.

### Induction of Posterior Foregut Cells

Human iPSCs were differentiated into foregut cells using previously described methods.^[^ ^6^ ^]^ In brief, iPSCs were detached by Accutase (Thermo Fisher Scientific) and were seeded on laminin laminin‐coated tissue culture plate with 100000 cells cm^−2^. Medium was changed to RPMI 1640 medium (Life Technologies) containing 100 ng mL^−1^ Activin A (Irvine Scientific) and 50 ng mL^−1^ bone morphogenetic protein 4 (BMP4; R&D Systems) at day 1, 100 ng mL^−1^ Activin A and 0.2% Knockout serum replacement (KOSR; Gibco) at day 2, and 100 ng mL^−1^ Activin A and 2% KOSR at day 3. On days 4 to 6, cells were cultured in Advanced DMEM/F12 (Thermo Fisher Scientific) with B27 (Gibco) and N2 (Gibco) containing 500 ng ml^−1^ fibroblast growth factor (FGF4, Peprotech) and 3 µM CHIR99021 (R&D Technologies). Cells were maintained at 37 °C in 5% CO2 with 95% air, and the medium was replaced every day. The foregut cells were detached by Accutase and seeded into Gri3D plates or used to generate standard HLO cultures in Matrigel dome.

### Generation of HLO Microarray Culture

Foregut cells were dissociated using Accutase, centrifuged at 500g rpm for 5 min at 4 °C, and resuspended in organoid enrichment medium. This medium consisted of Advanced DMEM/F12 supplemented with 3 uM CHIR99021, 5 ng/ml FGF2, (R&D Technologies), 10 ng ml^−1^ vascular endothelial growth factor (VEGF), (Life Technologies), 20 ng ml^−1^ epidermal growth factor (EGF), (R&D Technologies), 0.5 uM A83‐01, (Tocris), 50 ug ml^−1^ Ascorbic Acid, (Sigma), and 2% Matrigel (Corning). The resulting cell suspension was seeded into Gri3D plates (500 µm microcavities, Sun Biosciences, Switzerland) at a density of 250 cells/50uL per microcavity. After allowing cells to settle, 130 µL of organoid enrichment medium without Matrigel was added through the media exchange port. Cultures were incubated at 37 °C. After four days, the medium was replaced with liver specification medium consisting of Advanced DMEM/F12 supplemented with 2 µM retinoic acid (RA, Sigma) and maintained for an additional four days. This was followed by nine‐day culture in liver maturation medium. The liver maturation medium was based on HCM media (Lonza), prepared according to the manufacturer's instructions, but without EGF, and supplemented with 100 nM Dexamethasone (Sigma), 20 ng ml^−1^ recombinant human oncostatin M (Peprotech), and 10 ng ml^−1^ recombinant human hepatocyte growth factor (HGF) (Peprotech). HLO microarray cultures were maintained at 37 °C till they were fully matured. Media changes were performed every two days during the organoid formation and specification phases and daily during the liver maturation stage.

### Generation of Conventional HLO Culture

Foregut cells released using Accutase were centrifuged at 500g for 5 min, resuspended in Matrigel (Corning), and cultured using the previously described method with modifications.^[^ ^6^ ^]^ Briefly, A total of 100000 cells were embedded in 50µl Matrigel drop on the dishes in organoid formation media with 5 factors for 4 days. After organoid formation, the media were switched to liver specification media for 4 days. After the liver specification step, organoids were harvested from Matrigel by scratching and pipetting. Then, organoids were re‐embedded in Matrigel and grown in liver maturation media for an additional 9 days till they reached a full maturation state.

### Immunostaining

HLOs were fixed in 4% paraformaldehyde (Wako) in phosphate‐buffered saline overnight at 4 °C. Samples were permeabilized with 0.5% Triton X‐100 in 1×PBS, blocked with donkey serum (Millipore), and probed with primary antibodies against albumin (Bethyl) and Vimentin (Abcam) at 4 °C for 48h. Samples were probed with secondary antibodies conjugated with Alexa Fluor (Life Technologies) and DAPI (Sigma‐Aldrich) for nuclear staining. Images were acquired using ImageXpress Micro Confocal High‐Content Imaging System (Molecular Devices).

### Immunohistochemistry

Paraffin‐embedded tissue sections were deparaffinized, rehydrated, and boiled to retrieve antigens (10mM citrate buffer, pH = 6) under heat‐induced conditions, followed by two washes in PBS. Sections were blocked with 10% Normal Donkey Serum (NDS) in PBS‐T (PBS/0.1% Triton X‐100) for 1h at RT. Primary antibodies were hand‐applied and incubated for 32 min. After incubation, slides were treated with Multimer HRP, followed by the application of one drop of OmMap anti‐Mouse HRP for 20 min. Detection was visualized using DAB chromogen, and sections were counterstained with Hematoxylin II for 8 min, followed by application of a bluing reagent for 4 min. Finally, slides were washed in PBS, dehydrated, and mounted with ProLong Gold Antifade Mountant (Invitrogen). For imaging, slides were analyzed using a Leica DM18. Antibodies used are listed in the key resources table.

### Gene Expression Analysis

RNA was extracted and purified by Quick‐RNA miniprep kit (Zymo Research, cat. R1051) following the manufacturer protocol. Briefly Matrigel embedded and Gri3D grown organoids were washed twice in PBS. Organoids were submerged in RNA lysis buffer, and debris was removed by centrifugation prior to purification. RNA concentration and purity was assesed by Nanodrop (Thermofisher). CDNA was synthesized using 200 ng total RNA using a Superscript IV VILO synthesis kit (Thermofisher). PCR was performed using TaqMan gene expression master mix (Applied Biosystems) on a QuantStudio 6 Flex Real‐Time PCR System (Thermo, Applied Biosystems). All primers and probes used are listed in the key resource table.

### Biochemical Assays

Culture supernatants were analyzed to quantify albumin and CK18 levels using sandwich ELISA kits (Albumin, Abcam, and M65 EpiDeath CK18Diapharma) according to manufacturer's protocols. TNFα and Granzyme B secretion were measured using the Luminex Discovery Assay kit (R&D Systems/Biotechne) according to the manufacturer's protocol.

### Cytotoxicity Assessment in HLO Microarrays

Chlorpromazine (Sigma) and Streptomycin (MCE) were dissolved in DMSO, and 5–7 serial dilutions of 200 × stock solutions were prepared alongside corresponding vehicle controls (0.5% DMSO). Aliquots were frozen to ensure consistency across experiments. On the day of treatment, aliquots were thawed and diluted in liver maturation medium to achieve final working concentrations. Flucloxacillin (Sigma) was freshly prepared in liver maturation medium immediately prior to each dosing. HLO microarrays were treated over a 7‐day period, with a single re‐dosing and media refreshment on day 3. The highest concentrations tested for all compounds were 1000 µM. These exposure levels provide sufficient margins to support interpretation of observed hepatotoxic effects in the context of clinically relevant dosing.

### HLO viability Assay

Cell viability was assessed on day 7 using the CellTiter‐Glo 3D Cell Viability Assay (Promega, Cat. G9682). Luminescence was recorded using a Spectra Max iD3 microplate reader (Molecular Devices) following the manufacturer's recommended settings. Viability data from compound‐treated HLOs were normalized to the corresponding vehicle controls on the same plate.

### Dose‐Response Analysis and Margin of Safety (MOS) Calculation

Dose‐response curves and IC_50_ values were determined by nonlinear regression of log‐transformed compound concentrations (6‐8 point serial dilutions including vehicle) against normalized viability data. Curve fitting was performed using a variable Hill slope and constraining the top and bottom of the curve to 100 and 0, respectively (GraphPad Prism, GraphPad Software, La Jolla, CA, USA). To contextualize in vitro toxicity data with human exposure levels, Margin of Safety (MOS) values were calculated as the ratio of the in vitro IC_50_ to the compound's reported clinical maximum plasma concentration (MOS = IC_50_ / Cmax).

### Image Analysis and Quantification

Acquired brightfield images were processed using INCarta high‐content image analysis software (Molecular Devices) and a deep learning‐based model, SINAP, pretrained with thousands of images of stem cells and organoids. SINAP allows the user to define on‐demand objects of interest specific for the use case and learns from the parameters captured in the training dataset (e.g., morphology, texture, intensity, etc.) to further finetune the hyperparameters of the pretrained model enabling accurate and fast image segmentation. A SINAP module was hence trained by encircling the boundaries of HLOs grown in Gri3D and Matrigel plates as signal, while the background was defined by marking regions not covered by HLOs. To ensure the inclusiveness of different morphologies, HLO images was utilized from different donors, batches, and morphologies in training this SINAP model. SINAP‐generated masks were visually compared to raw images and demonstrated high accuracy in segmenting HLOs. The number and size of the organoids were then analyzed using in‐house written python scripts.

### PBMC Isolation and DC Differentiation

Peripheral blood mononuclear cells (PBMCs) were isolated from blood samples using density gradient centrifugation (SepMate‐50, STEMCELL Technologies). The whole blood was diluted with an equal volume of DPBS (Gibco) and layered over density gradient medium (Ficoll Paque Plus, Cytiva). The PBMCs were collected, washed with DPBS, and resuspended in X‐Vivo15 medium (Lonza). Monocytes were isolated by adherence from PBMCs cultured for 3 h in DC medium (X‐Vivo supplemented with 1% penicillin/streptomycin and 2% human serum (ThermoFisher) and differentiated into dendritic cells (DCs) using a cocktail of 100 IU/ml IL‐4 and 800 IU mL^−1^ GM‐CSF (Peprotech) for 4 days.

### Naïve CD8^+^ T Cell Isolation and Priming

Naïve CD8⁺ T cells were isolated from PBMCs using the EasySep Human Naïve CD8 T Cell Isolation Kit II (STEMCELL Technologies) according to the manufacturer protocol. Isolated CD8^+^ T cells were resuspended in X‐Vivo15 media supplemented with 1% penicillin/streptomycin (ThermoFisher) and 50 IU/ml IL‐7 (Peprotech) and incubated at 37 °C for 4 h. For the initial priming, immature DCs were matured and activated with 1600 IU/ml GM‐CSF, 100 IU ml^−1^ IL‐4, 100 IU mL^−1^ IFN‐γ (Peprotech), and 50 ng/ml LPS (InvivoGen). DCs were pulsed with Flucloxacillin at 1000 µM (Flux loading) or media alone as a mock condition. Naïve CD8^+^ T cells were added to Mock/Flux‐loaded DCs in T cell priming medium consisting of DC medium supplemented with 50 IU/ml IL‐21, 25 IU mL^−1^ IL‐12 (Peprotech) at a 3:1 T cell‐to‐DC ratio. After three days, the medium was replaced with T cell growth medium containing DC medium with 50 IU/ml IL‐7, 50 IU ml^−1^ IL‐15 (Peprotech), and the cells were cultured for an additional nine days, bringing the total culture time to 12 days. For the second priming, fresh DCs were prepared and loaded as described above. T cells were collected and co‐cultured with the freshly prepared DCs at a 5:1 T cell‐to‐DC ratio for two days in DC medium supplemented with 10 IU/ml IL‐7, 10 IU mL^−1^ IL‐15. T cell antigen‐specific activation efficacy was evaluated using multi‐color flow cytometry with Diva and FlowJo (BD software). T cell proliferation and activation were assessed using CFSE (1 µM, ThermoFisher) for proliferation, Human TruStain FcX for Fc receptor blocking, and a panel of antibodies including CD3 (clone UCHT1), CD8 (clone 3B5), CD4 (clone RPA‐T4), CD45RO (clone UCHL1), CD107a (clone H4A3), HLA‐DR (clone TU36), CD137 (clone 4B4), and CD69 (clone FN50). All reagents were used according to manufacturer's recommendations and are detailed in the key resource table. The strength of the response was graded according to the stimulation index (Supporting Information). The stimulation index is defined as a fold‐change increase in the CFSE^low^ (divided) or T cell activation marker‐positive cell population after priming with the Flucloxacillin relative to the CFSE^high^ (undivided) marker‐positive cells in non‐primed population (medium alone).

### HLO and T cell co‐Culture

Following initial 12 day priming as described above, CD8^+^ T cells were stained with CFSE (5 µM, Fisher Scientific, Catalog No. 11‐0699‐42) and resuspended in an optimized co‐culture media. This media consisted of a 1:1 mixture of complete RPMI (RPMI + 1% penicillin/streptomycin and 150 IU mL^−1^ IL‐2) and modified liver maturation media (excluding HGF, dexamethasone and hydrocortisone) supplemented with 10 IU mL^−1^ IL‐7 and 10 IU/ml IL‐15. Labeled CD8⁺ T cells were added to mature (day 23) HLO microarray cultures at effector‐to‐target (E:T) ratios of 1:1 and 1:5. These ratios were chosen to reflect the localized enrichment of cytotoxic lymphocytes observed during immune‐mediated liver injury, including periportal CD8⁺ T cell infiltration in flucloxacillin‐induced DILI (PMID: 24 731 753).^[^ ^14^ ^]^ Prior to co‐culture, HLO were pretreated with 100 µM Flucloxacillin for 72 h. The co‐culture was maintained for 72 h with media exchanges performed every 24 h. When indicated, T cell activation was induced using anti‐CD3 (1 µg ml^−1^, BioLegend) and anti‐CD28 (1 µg ml^−1^, BioLegend) antibodies.

### Quantification and Statistical Analysis

Statistical analyses were carried out using GraphPad Prism 10.0 (GraphPad Software,Inc., CA, USA). Group sizes, definition of error bars, and statistical analysis performed are indicated in figure legends. Unless otherwise noted, each experimental condition per donor was performed using 6–8 replicate Gri3D microwells and repeated in at least three independent experiments.

## Conflict of Interest

The authors declare no conflict of interest.

## Author contributions

F.E.A.S. and M.B. are co‐first authors and contributed equally to this work. Conceptualization, M.K. and A.R; Methodology, F.E.S., M.B, E.B., S.D., R.B., A.F., A.R., and M.K.; Formal analysis, F.E.S., M.B, S.D., M.K.; Investigation, F.E.S., M.B, E.B., W.C.B., S.D.; Visualization, F.E.S. M.B., S.D., E.B., and M.K.; Writing – original draft, F.E.S. and M. K.; Writing – Review & Editing, F.E.S., M.B., R.B., V.M.D., M.H., T.T, A.R., and M. K; Supervision, A.R. and M.K.; Project Administration, M.K.; Funding Acquisition, M.H., A.R., and M.K.

## Supporting information



Supporting Information

## Data Availability

The data that support the findings of this study are available from the corresponding author upon reasonable request.
