# Supplementary material for: Autologous Organoid‐T Cell Co‐Culture Platform for Modeling of Immune‐Mediated Drug‐Induced Liver Injury
Source: Adv Sci (Weinh). 2025 Sep 26;12(43):e08584. doi: 10.1002/advs.202508584 (PMC12631937; doi:10.1002/advs.202508584)
Supplement: Supplementary file 1 — Supporting Information [file ADVS-12-e08584-s001.docx]

Supporting Information

**Autologous Organoid-T Cell Co-Culture Platform for Modeling of Immune-Mediated Drug Induced Liver Injury**

*Fadoua El Abdellaoui Soussi^1^, Michael Brusilovsky^1^, Emma Buck^1^, W Clark Bacon^1^, Sina Dadgar^1^, Aaron Fullerton^2^, Victoria Marsh Durban ^3^, Riccardo Barrile^1,4^, Michael A. Helmrath^1^, Takanori Takebe^1,5,6^, Adrian Roth^7^, Magdalena Kasendra^1^*

**Table S1. Human iPSC lines used in this study.**

| No | Line ID | Age^†^ | | | Sex^†^ | Race^†^ | HLA Haplotype Class I |
| --- | --- | --- | --- | --- | --- | --- | --- |
| 1 | 72.3; Donor 1 | # | | | Male | # | A*02:01/A*31:01-B*40:01/B*49:01 C*03:04/C*07:01 |
| 2 | 461 | 50 | | | Male | Asian | A*68:01/A*03:01-B*35:03/B*35:03-C*12:03/C*04:01 |
| 3 | 470; Donor 2 | 50 | | | Female | African American | A*23:17/A*33:01-B*35:01/B*78:01-C*04:01/C*16:01 |
| 4 | 522 | 30 | | | Male | White | A*02:01/A*03:01-B*07:02/B*51:01-C*04:01/C*07:02 |
| 5 | 524; Donor 3 | 35 | | | Male | Asian | A*24:02/A*26:01-B*40:02/B*52:01-C*03:04/C*12:02 |
| 6 | 534 | 30 | | | Female | Asian | A*01:01/A*31:01-B*15:05/B*57:01-C*03:03/C*06:02 |
| 7 | 622 | | 51 | Male | | White | A*02:01/A*29:02-B*07:02/B*57:01-C*06:02/C*07:02 |
| 8 | 650 | | 31 | Male | | White | A*02:01/A*68:01-B*44:02/B*57:01-C*05:01/C*06:02 |
| 9 | 670 | | 26 | Male | | White | A*23:01/A*68:01-B*07:02/B*40:27-C*03:04/C*15:05 |
| 10 | 681 | | 50 | Female | | White | A*01:01/A*02:01-B*38:01/B*57:01-C*06:02/C*12:03 |

^†^ Self-declared

\# No data available

**A**

Donor 3

Donor 1

Donor 2

**Matrigel**

**Gri3D**

**C**

**B**

**500µm**

**Figure S1. Gri3D Microarrays Improve Organoid Homogeneity and Reproducibility Across Donors, related to Figure 1 (A)** Representative brightfield images of organoid cultures grown as Matrigel domes and Gri3D microarrays across three iPSC donors at day 23. Scale bar, 500 µm. **(B)** Quantification of intra-donor variability in organoid count and diameter, expressed as the coefficient of variation (CV), demonstrating improved uniformity of parallel samples from the same line in Gri3D cultures compared to Matrigel domes. (n = 32-36). **(C)** Quantification of inter-donor variability in organoid count and diameter, showing reduced line-to-line variation in Gri3D-grown HLOs compared to Matrigel-based cultures (n = 3 donors).

**Figure S2. Chlorpromazine Induces Intrinsic Hepatotoxicity Across Multiple HLO Lines, While Flucloxacillin and Streptomycin Show No Toxicity at Clinically Relevant Exposures.
(A–C)** Quantification of cell viability (% relative to vehicle-treated controls) following seven-day treatment of HLO microarrays derived from three independent iPSC donor lines with Chlorpromazine **(A),** Flucloxacillin **(B**), or Streptomycin **(C).** Data represent mean ± SEM from n = 4–8 technical replicates per concentration. Representative of three biologically independent experiments is shown, each using HLOs derived from a different iPSC donor line**.**

**Figure S3. HLA-B*57:01–Restricted CD8⁺ T Cell Activation Following Flucloxacillin Priming, related to Figure 3. (A)** Representative flow cytometry plots illustrating the expression of CD8⁺ T cell activation markers in response to dendritic cell (mDC) priming with Flucloxacillin (Flux) or mock (media alone). Data are shown for one HLA*B57:01 carrier and one HLA*B57:01 non- donor (control).
